# High-Density Lipoprotein Particles, Inflammation, and Coronary Heart Disease Risk

**DOI:** 10.3390/nu17071182

**Published:** 2025-03-28

**Authors:** Eveline O. Stock, Bela F. Asztalos, John M. Miller, Lihong He, Kate Townsend Creasy, Rachel Schwemberger, Alexander Quinn, Clive R. Pullinger, Mary J. Malloy, Margaret R. Diffenderfer, John P. Kane

**Affiliations:** 1 Cardiovascular Research Institute (CVRI) and Department of Medicine, University of California, San Francisco, CA 94143, USA; creasykt@upenn.edu (K.T.C.); rschwemberger@alamedahealthsystem.org (R.S.); alexander.quinn@pennmedicine.upenn.edu (A.Q.); clive.pullinger@ucsf.edu (C.R.P.); mary.malloy@ucsf.edu (M.J.M.); john.kane@ucsf.edu (J.P.K.); 2 Cardiovascular Research Laboratory, Human Nutrition Research Center on Aging, Tufts University, Boston, MA 02111, USA; bela.asztalos7@gmail.com; 3 J2 Health, New York, NY 10006, USA; john@j2health.com; 4 Boston Heart Diagnostics, Framingham, MA 01702, USA; lhe@bostonheart.eurofinsus.com (L.H.); margaret.diffenderfer@bostonheart.eurofinsus.com (M.R.D.); 5 Biobehavioral Health Sciences, PenNSAM—Penn Center for Nutrition Science and Medicine, Institute for Diabetes, Obesity, and Metabolism, Philadelphia, PA 19104, USA; 6 Department of Pediatrics, Alameda Health System, Highland Hospital, Oakland, CA 94602, USA; 7 Department of Hospital Medicine, Hospital of the University of Pennsylvania, Philadelphia, PA 19104, USA

**Keywords:** HDL, inflammation, CHD

## Abstract

Background: Coronary heart disease (CHD) remains a leading cause of death and has been associated with alterations in plasma lipoprotein particles and inflammation markers. This study aimed to evaluate and compare standard and advanced lipid parameters and inflammatory biomarkers in CHD cases and matched control subjects. We hypothesized that incorporating advanced lipid and inflammatory biomarkers into risk models would improve CHD risk prediction beyond the standard lipid measures. Methods: CHD cases (*n* = 227, mean age 61 years, 47% female) and matched controls (*n* = 526) underwent fasting blood collection while off lipid-lowering medications. Automated chemistry analyses were performed to measure total cholesterol (TC), triglycerides (TGs), low-density lipoprotein-C (LDL-C), small dense LDL-C (sdLDL-C), apolipoproteins (apos) A-I and B, lipoprotein(a) (Lp(a)), high-sensitivity C-reactive protein (hsCRP), serum amyloid-A (SAA), myeloperoxidase (MPO), and apoA-I in HDL particles (via 2-dimensional electrophoresis and immunoblotting). Univariate, multivariate, and machine learning analyses compared the CHD cases with the controls. Results: The most significant percent differences between male and female cases versus controls were for hsCRP (+78%, +200%), MPO (+109%, +106%), SAA (+84%, +33%), sdLDL-C (+48%; +43%), Lp(a) (+43%,+70%), apoA-I in very large α-1 HDL (−34%, −26%), HDL-C (−24%, −27%), and apoA-I in very small preβ-1 HDL (+17%; +16%). Total C, non-HDL-C, and direct and calculated LDL-C levels were only modestly higher in the cases. Multivariate models incorporating advanced parameters were statistically superior to a standard model (C statistic: men: 0.913 vs. 0.856; women: 0.903 versus 0.838). Machine learning identified apoA-I in preβ-1-HDL, α-2-HDL, α-1-HDL, α-3-HDL, MPO, and sdLDL-C as the top predictors of CHD. Conclusions: This study introduces a novel approach to CHD risk assessment by integrating advanced HDL particle analysis and machine learning. By assessing HDL subpopulations (α-1, α-2, preβ-1 HDL), inflammatory biomarkers (MPO, SAA), and small dense LDL, we provide a more refined stratification model. Notably, preβ-1 HDL, an independent risk factor reflecting impaired cholesterol efflux from the artery wall, is highlighted as a critical marker of CHD risk. Our approach allows for earlier identification of high-risk individuals, particularly those with subtle lipid or inflammatory abnormalities, supporting more personalized interventions. These findings demonstrate the potential of advanced lipid profiling and machine learning to enhance CHD risk prediction.

## 1. Introduction

Current coronary heart disease (CHD) risk assessment, as recommended by the American Heart Association (AHA), includes parameters such as age, sex, total cholesterol, high-density lipoprotein cholesterol (HDL-C), lipid-lowering treatment, systolic blood pressure, blood pressure treatment, body mass index (BMI), estimated glomerular filtration rate, diabetes, and smoking [1,2]. The latest AHA/American College of Cardiology (ACC) cholesterol guidelines highlight triglycerides > 175 mg/dL, lipoprotein(a) (Lp(a)) > 50 mg/dL or > 125 nmol/L, and high-sensitivity C-reactive protein (hsCRP) > 2.0 mg/L as CVD risk enhancers warranting aggressive lipid-lowering therapy [3]. In addition to hsCRP, other inflammation markers such as myeloperoxidase (MPO) and serum amyloid A (SAA) are associated with increased CHD risk [4,5,6,7,8,9,10].

Clinical trial evidence strongly supports lowering low-density lipoprotein cholesterol (LDL-C) through lifestyle modifications, statins, ezetimibe, and proprotein convertase subtilisin/kexin type 9 (PCSK9) inhibitors [11]. Direct measurements of LDL-C are superior to calculated LDL-C for CHD risk assessment [12]. Small dense LDL (sdLDL) particles (separated by electrophoresis), historically linked to increased CHD risk [13,14], have recently been identified as the most atherogenic lipoprotein parameter in the Framingham Offspring Study and as a significant CHD risk enhancer beyond standard risk factors in the Pooling Project using automated chemistry analyses [15,16]. Importantly, statin therapy effectively normalizes sdLDL-C levels [17].

Low HDL-C is also recognized as a CHD risk factor [3,18]. However, HDL-C levels are less predictive of CHD compared with elevated LDL-C levels, largely because HDL function is not reliably reflected by its concentration. This discrepancy has prompted the development of methodologies aimed at assessing HDL particles as well as HDL function in promoting cellular cholesterol efflux capacity (CEC) [18,19,20,21,22,23,24,25]. Studies have consistently demonstrated that HDL functionality, rather than HDL-C levels, was a better predictor of CHD risk [21,22]. Interestingly, the JUPITER trial revealed that HDL particle number, as measured by nuclear magnetic resonance, was a superior predictor of CHD than HDL functionality [23]. A few laboratories have carefully examined HDL particle concentrations using electrophoresis or ultrafiltration, as well as their lipid and protein compositions, to better understand their associations with HDL functionality and CHD risk [19,24].

Given the known associations between lipoprotein particle composition, inflammatory markers, and CHD, we hypothesized that advanced lipid parameters, including HDL subfractions, small dense LDL-C, and key inflammatory biomarkers such as MPO and SAA, provide superior risk stratification for CHD compared with standard lipid metrics. This study aimed to evaluate these parameters in CHD cases and matched controls and assess their additive predictive value through multivariate and machine learning approaches.

Our primary objective was to examine the clinical utility of HDL particle analysis by quantifying apolipoprotein (apo) A-I content across the five major HDL particles, separated by two-dimensional gel electrophoresis following by immunoblotting, in men and women with coronary heart disease (CHD). These findings were compared with age- and sex-matched controls. Additionally, we measured direct LDL-C, sdLDL-C, and Lp(a) as well as plasma markers of inflammation, including hsCRP, MPO, and SAA. Univariate, multivariate, and machine learning analyses were used to comprehensively assess case control differences.

## 2. Materials and Methods

### 2.1. Study Design and Population

This case-control study included patients with established CHD selected from the Genomic Resource of the Cardiovascular Research Institute at the University of California San Francisco (UCSF), enrolled between 1999 and 2014. All samples were stored at −80 degrees without thawing until analysis. CHD cases were defined by fulfilling at least one of the following American College of Cardiology criteria: (1) history of angina pectoris or myocardial infarction (MI), (2) coronary atherosclerosis documented by coronary angiography, (3) post angioplasty, and/or (4) post coronary artery bypass grafting. A total of 227 subjects (median age 61 years; 47% female) met these criteria. Additionally, 528 healthy controls (46% female), matched by sex and age, with no history of CHD or diabetes and active in a regular exercise program at the time of enrollment, were included. All subjects were off lipid-lowering medications and fasting at sample collection, and only complete data sets were included, resulting in 753 eligible samples. The UCSF Committee on Human Research approved the study protocol, and written consent was obtained from all participants.

Measurements were performed on stored plasma samples drawn after an overnight fast into EDTA-containing tubes and immediately placed on ice to stop enzymatic reactions. Data on blood pressure, past medical history, and use of medications and supplements was obtained from our existing database. This information is provided in Table 1 and Table 2. In this study 92.0% of controls and 71.4% of cases were Caucasian (*p* < 0.001).

### 2.2. Laboratory Measurements

Plasma levels of total cholesterol, triglyceride (TG), and HDL-C and creatinine and uric acid were determined by standard enzymatic methods, as previously described, using Roche reagents on COBAS automated chemistry analyzers [26]. Direct LDL-C, sdLDL-C, and Lp(a) were also measured using assay kits obtained from Denka-Seiken (Niigata, Japan) on Roche COBAS automated chemistry analyzers (Roche Diagnostics, Indianapolis, IN) [12,15,26]. Non-HDL-C and VLDL-C were calculated as follows: non-HDL-C = (total cholesterol − HDL-C); VLDL-C = (total cholesterol − (LDL-C + HDL-C)). LDL-C was also calculated using the Friedwald formula: total cholesterol − HDL-C − (TG/5) [27]. Plasma apoA-I, apoB, Lp(a), hsCRP, MPO, and SAA were measured by immunoassays on an automated chemistry analyzer, as previously described [26]. The MPO immunoassay was obtained from Diazyme Laboratories (Poway, CA, USA) For all assays the within- and between-run coefficients of variation were < 5.0%.

The apoA-I content of HDL was analyzed by two-dimensional gel electrophoresis followed by immunoblotting [24]. The within- and between-run coefficients of variation were <10% for all particles except for preβ1, which was <15%. ApoA-I concentration was calculated by multiplying the percent distribution of apoA-I in the individual subparticle by the total plasma apoA-I concentration [24]. All analyses were performed in a blinded fashion.

### 2.3. Statistical Analysis

Descriptive data in Table 1 and Table 2 are expressed as median values with the interquartile range (IQR) based on the 25th and 75th percentile values. Categorical variables are reported as frequencies and percentages. Significance between groups was determined by chi-squared analysis for categorical variables and by non-parametric Kruskal–Wallis test for numerical variables. HsCRP, Lp(a), MPO, and TG were log-transformed prior to analysis because these variables did not have a normal distribution. Multivariate logistic regression analyses (see Table 3 and Table 4) were performed to identify parameters significantly associated with the presence or absence of CHD. These results are expressed as odds ratios for differences between cases and controls equal to a 1 mg/dL change in the parameter. C index analysis was used to determine if additional biomarkers improved prediction of CVD case status. All statistical analyses were performed using R software. A *p* value < 0.05 was considered statistically significant. Spearman correlation coefficient analysis between biochemical parameters is shown in Supplementary Tables S1 and S2. Coefficients of > 0.20 were statistically significant (*p* < 0.05).

### 2.4. Machine-Learning Analysis

The machine learning method of Random Forest classification was applied to the dataset. Random Forest classified models performed well on the classification task of identifying CHD cases versus control subjects, and the key decision tree variables were analyzed using Note Purity scores. The following Python (version 3.9) libraries were imported into the notebook environment to conduct analysis: numpy, pandas, matplotlib.pyplot, and sklearn. In this analysis, only biochemical parameters were utilized.

## 3. Results

### 3.1. Standard Risk Factors

Table 1 and Table 2 provide demographic data and standard lipid analyses in male and female CHD cases (*n* = 227) compared with healthy control subjects (*n* = 528). Men and women with CHD were somewhat younger, had higher BMI, significantly higher prevalence rates of hypertension and diabetes than controls, and more current cigarette smoking than control subjects.

Male CHD cases had only 1.3% higher median total cholesterol values, but significantly higher TG (+19.8%) and non-HDL-C values (+11.6%), as well as significantly lower HDL-C levels (−24.0%), compared with controls (Table 1). In addition, calculated LDL-C values were only 1.6% higher in male cases than controls and only 1.4% higher in female CHD cases versus controls. As in male cases, female cases had significantly higher TG (+29.7%) and non-HDL-C values (+17.1%), and significantly lower HDL-C levels (−26.9%), compared with controls (Table 2). Calculated LDL-C values were 11.7% higher in female cases than controls. The greatest percentage difference in lipid levels between CHD cases and controls was in HDL-C. HDL-C concentrations were substantially lower in CHD cases for both sexes.

### 3.2. Advanced Biochemical Risk Factors

Substantially greater differences between CHD cases and controls were noted when advanced testing parameters were examined. Male cases (Table 1) had only 2.6% higher median direct LDL-C values, but significantly higher sdLDL-C (+48.2%), % sdLDL-C of total direct LDL-C (+37.9%), VLDL-C (+24.0%), and Lp(a) values (+46.2%) compared with controls. Levels of apoB were only 6.8% higher (*p* = 0.007), and levels of apoA-I were only 9.8% lower. Much greater percent differences were seen with HDL particles. Male CHD cases had significantly (*p* < 0.001) lower levels of apoA-I in very large α-1 HDL (−34.2%), large α-2 HDL (−8.8%), and small α-4 HDL (−12.9%), and significantly higher levels of apoA-I in very small preβ-1 HDL (+16.7%). The largest percent differences in male cases versus controls were observed when inflammation markers were assessed. Male cases had significantly (*p* < 0.001) higher median values of MPO (+109%), SAA (+84.0%), and hsCRP (+77.8%) relative to controls.

Women with CHD also had substantially greater differences versus controls when advanced parameters were examined. As shown in Table 2, female cases had significantly higher direct LDL-C (+16.4%), sdLDL-C (+42.3%), % sdLDL-C (+7.8%), VLDL-C (+8.7%), and especially Lp(a) values (+69.8%) than controls. Levels of apoB were also significantly higher (+16.3%), and levels of apoA-I were significantly lower (−8.3%) when cases were compared with controls. Female CHD cases had significantly lower levels of apoA-I in very large α-1 HDL (−26.4%), large α-2 HDL (−8.8%), and small α-4 HDL (−5.6%), and significantly higher levels of apoA-I in very small preβ-1 HDL (+16.0%). As with men, the largest differences in female cases versus controls were seen with inflammation markers. Female cases had significantly higher median values of hsCRP (+200.0%), MPO (+106%), and SAA (+33.3%) compared with controls.

### 3.3. Multivariate Analysis

Multivariate modeling was performed to assess the differences in various parameters between CHD cases and healthy controls. Such analyses are limited because of correlations between variables. HDL-C was highly correlated with apoA-I in very large α-1 HDL and large α-2 HDL, and inversely correlated with TG levels (see Supplementary Tables S1 and S2). Total cholesterol and non-HDL-C were highly correlated with each other and with calculated LDL-C and direct LDL-C, but less strongly with sdLDL-C. HsCRP values were quite highly correlated with SAA values, and when SAA was added to the model with hsCRP already present, SAA was no longer significant.

Correlation coefficient analysis results (Spearman) for men and women are shown in Supplementary Tables S1 and S2. ApoA-I levels in preβ1 HDL in men and women were most strongly positively correlated with log sdLDL-C (0.412, 0.475), total cholesterol (0.395, 0.365), non-HDL-C (0.343, 0.365), and α-3 HDL (0.255, 0.312). ApoA-I levels in α-1 HDL in men and women were most strongly positively correlated with HDL-C (0.899, 0.903), α-2 HDL (0.708, 0.610), and inversely correlated with log TG (−0.475, −0.491) and non-HDL-C (−0.261, −0.363). All values > 0.20 were statistically significant (*p* < 0.05).

Results of the multivariate model are shown in Table 3 for men and in Table 4 for women. In the first model, which included HDL-C, non-HDL-C, hypertension, diabetes, and smoking, the C statistic or area under the curve (AUC) value for distinguishing cases from controls for men was 0.856 and for women was 0.838. When log hsCRP was added to this model, the AUC value increased to 0.859 in men and to 0.842 in women. With the further addition of log sdLDL-C and log Lp (a), the AUC value further increased in men and women to 0.869 and 0.844, respectively. Finally, when apoA-I in all HDL particles was added to the previous model in men and women, the AUC values increased to 0.913 and 0.903, respectively. These increases in AUC values for the models were all statistically significant (*p* < 0.01).

### 3.4. Machine Learning Analysis

Machine learning methods were applied to the male and female subset data. Each subset was split into training and testing data groupings, with 70% allocated to training and 30% for testing the model. The default Random Forest classifier with 100 estimators was applied to the training data, and it scored very highly in terms of training/testing accuracies. The male model had 100% accuracy on the training data and 92.3% accuracy on the testing data; the female model had 94.1% accuracy on the training data and 98.9% accuracy on the testing data. For men, there were 83 true negatives, 1 false positive, 7 false negatives, and 13 true positives; for women, there were 72 true negatives, 1 false positive, 2 false negatives, and 12 true positives. To interpret the mechanism behind the high predicting power of the models, we examined node purity scores. Each variable was given a purity score, or the relative importance it has to the model; the purities of the model were normalized such that they sum to 1. In both the male and female models, apoA-I in preβ-1 HDL and apoA-I in α-2 HDL were deemed to be the two most important variables for distinguishing cases from controls.

In men, the node purity scores from the Random Forest analysis based on the biochemical data for identifying CHD cases were as follows: (1) α-2 HDL 0.233, (2) preβ-1 HDL 0.185, (3) log MPO 0.172, (4) α-1 HDL 0.096, (5) sdLDL-C 0.095, (6) α-3 HDL 0.075, (7) creatinine 0.037, (8) uric acid 0.033, (9) log CRP 0.032, (10) log TG 0.021, (11) α-4 HDL 0.016, and (12) log Lp(a) 0.004. Of the total purity sum of 1.0, the amount associated with the HDL particles data sum for the biochemical variables was a striking 0.777, or about 78% of the total. The true positive rate was 76.9%, and the true negative rate was 98.9%.

In women, the node purity scores from the Random Forest analysis revealed the following results based on biochemical data for identifying CHD cases: (1) preβ-1 HDL 0.350, (2) α-2 HDL 0.151, (3) sdLDL-C 0.131, (4) log MPO 0.130, (5) α-3 HDL 0.047, (6) α-1 HDL 0.035, (7) uric acid 0.035, (8) log CRP 0.0228), (9) creatinine 0.027, (10) α-4 HDL 0.022, (11) log Lp(a) 0.020, and (12) log TG 0.018. Of the total purity sum of 1.0, the amount associated with the HDL particles data sum for the biochemical variables was 0.605, or about 60%. The true positive rate was 84.6%, and the true negative rate was 100.0%.

As a last step, a naïve Random Forest classifier was built with all numeric variables fed into it; without the clinician’s perspective on important factors, this was a validation step to see what the machine learning method found to be the most important variables without any help from a knowledgeable source. Achieving a 100% accuracy on training data and 90.6% accuracy on testing data, the naïve model also indicated that preβ-1 HDL and α-2 HDL were the most important bioinformatic readings to distinguish between CHD cases and controls.

## 4. Discussion

Traditional coronary heart disease (CHD) risk assessment relies on measuring serum total cholesterol, triglycerides, and HDL-C, typically after precipitation of other lipoproteins in the lipid profile analysis [1,2,3,12]. While some laboratories also measure serum apoA-I, the primary protein of HDL, current evidence does not support its added value over HDL-C for CHD risk prediction [26]. In addition, a much smaller number of laboratories assess HDL particle number by nuclear magnetic resonance, with evidence suggesting this measurement provides enhanced predictive capability for CHD risk assessment beyond HDL-C [23].

The role of high-density lipoprotein (HDL) particles in CHD risk has long been a central focus of research, particularly regarding the functional properties of these particles rather than their concentrations alone. Emerging evidence suggests that the functionality of HDL, especially specific subtypes like preβ-1 HDL, plays a much more significant role in disease pathogenesis. Preβ-1 HDL has become a critical marker in understanding vascular risk due to its close association with cholesterol efflux capacity—the process by which cholesterol is removed from the artery wall and transported back to the liver for excretion or recycling. This reverse cholesterol transport (RCT) process is a key protective mechanism in cardiovascular health and offers significant insights into CHD risk [21,22]. Recent advances in HDL proteomics and lipidomics have also highlighted the potential roles of HDL in inflammation, oxidation, and coagulation, expanding its relevance in CHD research [24,25,26,27,28,29].

The data presented here focus on the characterization and role of HDL particles containing apoA-I, encompassing five subtypes of HDL particles in plasma as assessed by two-dimensional gel electrophoresis [30]. These HDL subtypes range from very-small-discoidal preβ-1 HDL (5.6 nm in diameter, comprising about 6% of HDL particles in normal plasma) to very-large-spherical α-1-HDL (about 11.0 nm in diameter, comprising about 22% of HDL particles), which are important components of lipid metabolism and have been implicated in cardiovascular health. Other HDL particles include small-discoidal α-4-HDL (about 7.4 nm in diameter, comprising about 13% of HDL particles), medium-spherical α-3-HDL (about 8.0 nm in diameter, comprising about 15% of HDL particles), and large-spherical α-2-HDL (about 9.2 nm in diameter, comprising about 44% of HDL particles) [5,24,26,31].

**Figure 1:**
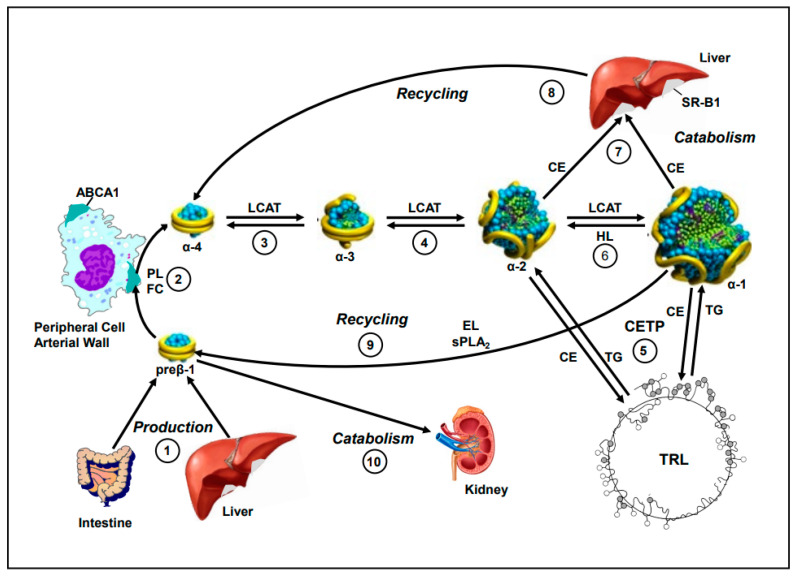
HDL Particle metabolism. After the production of very-small-discoidal lipid-poor preβ-1 HDL in the liver or intestine (step 1), these particles bind to cellular ABCA1, resulting in the removal of FC and PL from cells, including those in artery wall plaques (step 2), and the formation of small-discoidal α-4 HDL. These particles, in turn, are converted to medium-spherical α-3 HDL when their free cholesterol is esterified to CE by the action of LCAT. Moreover, efficient lipolysis via the action of lipoprotein lipase on TRL is essential to the formation of larger HDL particles and for normal HDL metabolism and the transfer of TRL lipid and apolipoprotein constituents to HDL (step 3). Medium α-3 HDL is, in turn, converted to large α-2 HDL with further cholesterol esterification via LCAT (step 4). These particles, in turn, pick up TG from TRL in exchange for CE via CETP to form very large α-1 HDL. There is also further cholesterol esterification via LCAT (step 5). These particles also pick up TG in exchange for CE via CETP with some conversion of α-1 to α-2 HDL via HL (step 6). Large and very large α-2 and α-1 HDL deliver cholesterol (mainly CE) to the liver via SR-BI with some catabolism of HDL particles (step 7). Moreover, there is a lot of recycling and exchange of HDL constituents between all HDL particles and TRL and recycling of HDL constituents to re-form preβ-1 and α-4 HDL, facilitated by the action of EL and sPLA2 (steps 8 and 9). The final step in HDL particle metabolism is the catabolism of preβ-1 HDL via the kidney (step 10). This latter process is enhanced in patients with hypertriglyceridemia [32,33]. Abbreviations: ABCA1, ATP-binding cassette transporter A1; apo, apolipoprotein; CE, cholesteryl ester; CETP, cholesteryl ester transfer protein; EL, endothelial lipase; FC, free cholesterol; HDL, high-density lipoprotein; HL, hepatic lipase; LCAT, lecithin–cholesterol acyltransferase; PL, phospholipid; sPLA2, secretory phospholipase A2; SR-B1, scavenger receptor class B type 1; TG, triglyceride; TRL, triglyceride-rich lipoprotein.

Studies by Asztalos et al. have shown that these particles vary in their ability to engage in cholesterol efflux, with preβ-1 HDL being the primary acceptor of free cholesterol from peripheral cells via the ATP-binding cassette transporter A1 (ABCA1), while the larger α-1 and α-2 particles are involved in bi-directional cholesterol transport through interaction with the liver via the scavenger receptor type B1 (SR-B1) [24,25]. These dynamic roles make HDL particle analysis a promising approach to refine CHD risk stratification. A diagram of HDL particle metabolism is shown in Figure 1.

The staff at two United States laboratories have contributed substantial research on HDL particles relative to CHD and cardiovascular disease (CVD) risk: the laboratories of Drs. Bela Asztalos and Ernst Schaefer and staff at Tufts University in Boston and the laboratory of Dr. John Kane and colleagues at the University of California, San Francisco. Asztalos and colleagues at Tufts University have significantly contributed to the characterization of these five major apoA-I-containing HDL subpopulations. They have shown that coronary heart disease (CHD) patients often exhibit significantly lower levels of α-1 HDL, the largest and most protective HDL particle, and higher levels of smaller HDL particles, especially preβ-1 HDL. For example, in a study of 76 CHD cases versus 79 controls, Asztalos et al. found that apoA-I levels in α-1 HDL were significantly lower, by 35%, while apoA-I levels in preβ-1 HDL were significantly higher, by 8% [34]. Further studies have reinforced this association, showing that increases in preβ-1 HDL and decreases in α-1 HDL are strongly correlated with the presence and progression of CHD [35,36].

The Framingham Offspring Study, involving 1446 male participants, reported that those with CHD had significantly lower apoA-I levels in α-1 HDL (−32%) and higher levels in smaller HDL particles (α-3 and preβ-1 HDL) (+29% and +16%, respectively) [36]. These alterations in HDL particle distribution were predictive of CHD risk, as each 1 mg/dL increase in α-1 HDL was associated with a 26% reduction in the odds of CHD [36]. This finding highlights the protective role of large HDL particles, further emphasizing the utility of HDL particle analysis in risk assessment. It is also important to note that changes to HDL sub populations can be caused by statin therapy [37] and weight changes [38,39].

John Kane and colleagues have made pivotal contributions to our understanding of preβ-1 HDL, specifically its role in CHD risk. Kane’s laboratory was the first to identify preβ-1 HDL and has documented that elevated levels of preβ-1 HDL, or an increased proportion of apoA-I in preβ-1 HDL, are associated with a higher risk of CHD [19,40]. For instance, in a cohort of 1255 individuals, those with CHD and myocardial infarction (MI) were more likely to have elevated levels of preβ-1 HDL, with top tertile values for apoA-I in preβ-1 HDL at 40.6%, compared with 30.1% in controls [40]. This association persisted even after adjusting for established cardiovascular risk factors, further underscoring the potential of preβ-1 HDL as a risk marker.

A key concept to highlight is that elevated preβ-1 HDL levels, which Kane and colleagues have consistently associated with increased CHD risk, reflect impaired cholesterol efflux from the arterial wall, which is an independent contributor to cardiovascular risk beyond elevated lipoprotein levels alone. This dysfunction suggests that preβ-1 HDL serves as a marker of reduced efficiency in reverse cholesterol transport, indicative of a risk of cholesterol accumulation in the arterial wall and heightened cardiovascular risk. Moreover, Stock et al. [32] and Pullinger et al. [41] demonstrated that elevated levels of preβ-1 HDL were strongly correlated with hyperlipidemia, particularly combined hyperlipidemia and hypertriglyceridemia. In their studies, individuals with high levels of preβ-1 HDL had significantly higher odds ratios for CHD and MI, even after adjusting for traditional risk factors. These findings highlight the importance of preβ-1 HDL as an independent predictor of cardiovascular events.

Recently, Asztalos and colleagues expanded on this work in 2022 with studies involving 534 CHD patients and 1076 age-, sex-, and BMI-matched controls. In a subset of 100 cases and 100 controls, ABCA1-CEC and SR-BI-CEC were measured in apoB-depleted serum [24]. They found that CHD patients had significantly higher concentrations of preβ-1 HDL particles (+43%) and ABCA1-CEC (+34%) compared with controls. Moreover, ABCA1-CEC was positively correlated with preβ-1 particle concentrations, triglycerides (TG), small dense LDL cholesterol (sdLDL-C), and LDL-C in both cases and controls. The concentration and functionality of preβ-1 particles (ABCA1-CEC/mg preβ-1) were also positively associated with preβ-1 particle levels, triglycerides, sdLDL-C, and LDL-C (r = 0.424).

In contrast, CHD patients exhibited significantly lower concentrations of α-1 (−22%), α-2 (−7%), and α-4 (−11%) HDL particles, while SR-BI-CEC remained similar (+6%) compared with controls. SR-BI-CEC was positively correlated with HDL-C, apoA-I, and large HDL particle levels. However, the functionality of large HDL particles (SR-BI-CEC/mg large particles) was significantly and positively correlated with the preβ-1/α-1 ratio, sdLDL-C, and triglycerides. These findings suggest that CHD patients have significantly higher concentrations of, but less functional, preβ-1 HDL particles in terms of cholesterol efflux capacity compared with controls. Additionally, TG-rich lipoproteins have a significant impact on HDL particle concentrations and functionality. A key limitation of these studies was that all CHD patients were on statin therapy, while none of the controls were receiving such treatment. Statins have been shown to affect HDL particle profiles, potentially confounding the interpretation of the results [42].

The findings of the present study are consistent with these previous observations. We observed significant decreases in apoA-I levels in α-1 HDL and significant increases in apoA-I in preβ-1 HDL in male and female CHD subjects compared with matched controls. These alterations, along with elevated levels of other lipoprotein markers (e.g., sdLDL-C) and inflammatory biomarkers (e.g., hsCRP, MPO, SAA), are in line with previous studies documenting the association between altered HDL particle profiles and CVD risk [4,5,6,7,8,9,10,15,16,18,24,25,28,29,33,34,35,36,40,41,42,43,44].

Our study also identified significant improvements in CHD risk prediction when advanced biochemical parameters, including HDL particle analysis and inflammation markers, were incorporated into multivariate models. Machine learning analysis revealed that HDL particle subpopulations, particularly preβ-1 HDL, α-2 HDL, and α-1 HDL, along with inflammatory markers such as MPO and sdLDL-C, were the most informative for distinguishing CHD cases from controls. These findings further support the utility of advanced lipid and inflammatory marker profiling in enhancing the accuracy of cardiovascular risk assessment.

The novelty of this study lies in its comprehensive assessment of HDL functionality and particle composition rather than relying solely on traditional markers like HDL-C or apoA-I. Additionally, by integrating machine learning, we analyze the combined impact of HDL subpopulations (α-1, α-2, and preβ-1 HDL), inflammatory biomarkers (MPO, SAA), and small dense LDL, providing a more refined and data-driven approach to CHD risk stratification. Notably, we highlight preβ-1 HDL as an independent risk factor reflecting the efficiency of cholesterol efflux from the artery wall, an often overlooked yet critical component of atheroprotection. Elevated preβ-1 HDL levels indicate impaired reverse cholesterol transport and increased cardiovascular risk. These findings emphasize the potential of advanced lipid profiling and machine learning to refine and improve CHD risk assessment.

### Sex Differences in Biomarker Profiles and Implications

Sex differences in HDL subtype composition have been well documented in large studies (*n* = 83,812 women and 110,774 men), with women typically exhibiting 30% higher HDL-C levels and 18% higher apoA-I, especially in the large α-1 and α-2 HDL particles compared with men [31]. Men and women have similar concentrations of apoA-I in the smaller HDL particles (α-3, α-4, and preβ-1) [31]. These findings are important for understanding the varying CHD risk profiles between the sexes, with elevated levels of large HDL particles in women associated with a more favorable lipid profile and lower cardiovascular risk.

Lipid measures, particularly HDL-C and non-HDL-C, remain relevant, but their contribution alone is less robust than when combined with more advanced markers like sdLDL-C, Lp(a), and apoA-I. The AUC for CHD prediction using HDL-C alone was 0.67 in men and 0.66 in women, whereas the combination of advanced markers improved the AUC to 0.81 in men and 0.83 in women. This highlights the limitations of traditional lipid measures in capturing the full complexity of CHD risk and underscores the importance of integrating more refined biomarkers to improve risk prediction.

Our study expands on these prior observations by examining the predictive value of apoA-I levels for CHD risk in men and women. The addition of apoA-I in HDL particles provides the greatest improvement in the predictive power for CHD in both men and women, with men showing a slightly stronger increase in prediction strength (AUC improvement: 0.72 to 0.79 in men vs. 0.71 to 0.78 in women). This suggests that the mechanistic role of apoA-I in reverse cholesterol transport and HDL function may be particularly relevant in male populations for cardiovascular risk assessment. The area under the receiver operating characteristic curve (AUC) derived from the ROC analysis demonstrated that apoA-I in preβ-1 HDL, a marker of cholesterol efflux from the artery wall, was a strong predictor of CHD risk in both sexes, with AUCs of 0.872 in men and 0.858 in women. This suggests a comparable predictive utility across sexes, despite underlying differences in HDL-C levels.

Inflammation markers (hsCRP, SAA, MPO) contribute significantly to improving predictive models, particularly in women, where the addition of inflammation markers has a stronger effect (AUC improvement: 0.71 to 0.81 in women vs. 0.72 to 0.78 in men). This aligns with prior findings that women exhibit a more robust inflammatory response, which may explain why inflammatory biomarkers are stronger predictors of CHD in female populations. Consequently, incorporating inflammatory biomarkers in cardiovascular risk prediction models for women may enhance risk stratification and early intervention strategies.

When compared with C-reactive protein (CRP), a widely used inflammatory biomarker for CHD risk, apoA-I in preβ-1 HDL demonstrated superior predictive performance in both men and women. The AUC for CRP was lower than that of apoA-I in preβ-1 HDL, with values of 0.791 in men and 0.804 in women. Notably, the predictive advantage of apoA-I in preβ-1 HDL over CRP was more pronounced in men (AUC difference of 0.081), suggesting that HDL functionality-related markers may provide greater discrimination of CHD risk in men compared with systemic inflammatory markers.

Sex-specific differences in biomarker prediction should be considered when assessing cardiovascular risk. Men seem to benefit more from lipid-focused models, while women see greater predictive power when inflammation markers are added. These distinctions emphasize the necessity for sex-specific risk prediction models that account for variations in lipid metabolism, inflammation, and HDL function.

The combination of multiple biomarkers, including lipid, inflammatory, and HDL particle measures, contributes toward optimizing CHD prediction in both sexes, but the relative importance of these markers varies between men and women. Future studies should explore sex-specific thresholds for these biomarkers to refine personalized risk assessment, if integrating these biomarkers into existing risk prediction models improves their clinical utility, and whether interventions targeting HDL functionality yield sex-specific benefits in CHD prevention.

The strength of the present study lies in the large number of biochemical parameters that were measured as well as the absence of lipid-lowering medication use among the participants, which allowed for a more accurate depiction of the natural HDL particle profile in CHD subjects. The novelty of this study lies in its comprehensive assessment of HDL functionality and particle composition rather than relying solely on traditional markers like HDL-C or apoA-I. Additionally, by integrating machine learning, we analyze the combined impact of HDL subpopulations (α-1, α-2, and preβ-1 HDL), inflammatory biomarkers (MPO, SAA), and small dense LDL, providing a more refined and data-driven approach to CHD risk stratification. Notably, we highlight preβ-1 HDL as an independent risk factor reflecting the efficiency of cholesterol efflux from the artery wall, an often overlooked yet critical component of atheroprotection. Elevated preβ-1 HDL levels indicate impaired reverse cholesterol transport and increased cardiovascular risk. These findings emphasize the potential of advanced lipid profiling and machine learning to refine and improve CHD risk assessment. While the detailed lipid particle analysis and inflammatory markers used in this study are not currently part of routine clinical practice, our findings highlight their potential for enhanced risk stratification in select patient populations, warranting further investigation into their clinical utility and cost-effectiveness. Future studies focused on larger, more diverse cohorts and cost-effectiveness analyses are needed to translate these findings into clinically applicable strategies.

Limitations of this study include (1) the relatively small sample size; (2) the higher percentage of non-Caucasian subjects in the CHD case group than controls (28.6% versus 8.0%), which could have affected the outcomes of our analysis, especially for Lp(a), which is known to be much higher in African Americans; and (3) the fact that controls were required to be non-diabetic at the time of sampling. These limitations should be considered when interpreting our findings. While our sample size allowed us to observe significant differences in biomarker profiles, it is relatively small, particularly when considering the heterogeneity of CHD populations. The study population may not be representative of all the population of individuals with CHD, and the cross-sectional design limits our ability to establish causality. Potential confounding factors such as lifestyle habits, medication use, and undiagnosed conditions may have influenced our results. While we attempted to control for some of these factors, for example, by analyzing subjects not on lipid-lowering therapy, residual confounding cannot be ruled out. Furthermore, the tests used in this study, such as detailed lipid particle analysis, are not currently part of routine clinical practice. This limits the immediate translational potential of our findings. While our study focused on select HDL subfractions and inflammatory markers, it excluded other potentially informative biomarkers, such as markers of myocardial injury (e.g., high-sensitivity troponin, hsTnT) or cardiac stress (e.g., *N*-terminal pro B-type natriuretic peptide, NT-proBNP). We recognize that these markers could provide a more comprehensive risk assessment as they have become integral to the diagnosis, treatment, and prognosis of cardiovascular diseases, particularly in the context of coronary heart disease [45,46]. The inclusion of hsTnT and NT-proBNP would have provided valuable insights into the extent of myocardial injury and its relationship with the lipid and inflammatory markers examined in this study. Future research should consider incorporating these markers to provide a more comprehensive assessment of cardiovascular risk and to better understand the interplay between traditional risk factors, inflammation, and myocardial injury. These limitations may have affected the magnitude of the observed associations and limited the generalizability of our findings. However, we believe that our study provides valuable preliminary data that can inform future research. Therefore, larger, multicenter, longitudinal studies are needed to validate these findings; assess their generalizability; determine the temporal relationship between biomarker levels and CHD risk; include more diverse populations, considering factors such as age, ethnicity, and comorbidities; and evaluate their clinical utility and cost-effectiveness.

## 5. Conclusions

In conclusion, the data presented in this manuscript underscore the importance of HDL particle analysis in improving CHD risk assessment. The studies by Drs. Asztalos, Kane, and their colleagues have been instrumental in advancing our understanding of HDL particle composition and its relationship to CHD risk. Our findings highlight the potential of integrating advanced biomarkers into existing cardiovascular risk assessment frameworks, offering a more personalized approach to risk stratification. By identifying individuals at higher risk, especially those with subtle lipid or inflammatory abnormalities; this enables earlier, targeted interventions such as lifestyle modifications or more aggressive management of modifiable risk factors.

A key novel aspect of our study is the inclusion of preβ-1 HDL as a marker of cholesterol efflux capacity (CEC) from the artery wall. CEC plays a protective role in atherogenesis and clinical ASCVD, and incorporating preβ-1 HDL may improve risk prediction, particularly in patients with normal or elevated HDL-C levels, which have not consistently correlated with outcomes in clinical trials. While additional prospective studies are needed to confirm these findings, they may pave the way for new diagnostic and therapeutic strategies to improve cardiovascular outcomes. Moving forward, larger prospective studies are needed to validate these findings and explore the potential of HDL particle analysis as a tool for early detection and risk stratification in cardiovascular disease.

## Figures and Tables

**Table 1:** Risk factors in men with established CHD compared with controls *.

Variable	Controls (*n* = 285)	CHD Cases (*n* = 120)	Percent Difference ^†^	*p* ^‡^
Standard Risk Factors				
Age, years	68.8 (11.0)	61.0 (18.3)	−11.3%	<0.001
BMI, kg/m^2^	25.7 (4.7)	27.1 (5.5)	+5.4%	0.002
Hypertension, %	24.9%	60.0%	+141.0%	<0.001
Diabetes, %	0.4%	30.0%	+7400%	<0.001
Current smoker, %	1.8%	4.2%	+133.3%	0.281
Lipids, mg/dL				
Total cholesterol	194.0 (45.0)	196.5 (67.0)	+1.3%	0.616
Triglycerides	124.0 (89.0)	148.5 (157.5)	+19.8%	<0.001
HDL-C	50.0 (20.0)	38.0 (19.0)	−24.0%	<0.001
Non-HDL-C	142.0 (43.0)	158.5 (76.5)	+11.6%	0.002
Calculated LDL-C	115.8 (42.6)	117.6 (67.0)	+1.6%	0.733
Advanced Risk Factors				
Hs-C-reactive protein, mg/L	0.9 (1.7)	1.6 (3.2)	+77.8%	<0.001
Serum amyloid A, mg/L	2.5 (2.8)	4.6 (6.4)	+84.0%	<0.001
Myeloperoxidase, pmol/L	197 (61)	411 (251)	+109%	<0.001
Direct LDL-C, mg/dL	116.0 (40.0)	119.0 (63.5)	+2.6%	0.683
sdLDL-C, mg/dL	28.0 (18.0)	41.5 (35.0)	+48.2%	<0.001
sdLDL-C, % of total LDL-C	24.0 (10.3)	33.1 (19.0)	+37.9%	<0.001
VLDL-C, mg/dL	25.0 (13.0)	31.0 (21.2)	+24.0%	<0.001
ApoB, mg/dL	96.0 (28.0)	102.5 (42.0)	+6.8%	0.007
Lp(a), mg/dL	10.6 (16.4)	15.5 (32.9)	+46.2%	0.031
Total apoA-I, mg/dL	148.1 (32.9)	133.6 (34.2)	−9.8%	<0.001
ApoA-I in preβ-1 HDL, mg/dL	11.4 (5.2)	13.3 (7.1)	+16.7%	0.003
ApoA-I in α-4 HDL, mg/dL	17.8 (5.1)	15.5 (4.6)	−12.9%	<0.001
ApoA-I in α-3 HDL, mg/dL	23.6 (5.5)	22.6 (6.5)	−4.2%	0.059
ApoA-I in α-2 HDL, mg/dL	55.7 (15.5)	50.8 (16.5)	−8.8%	<0.001
ApoA-I in α-1 HDL, mg/dL	26.9 (16.7)	17.7 (14.3)	−34.2%	<0.001

* Data are expressed as median (interquartile range), except where percent is indicated. ^†^ CVD cases versus controls. ^‡^ *p* for trend across subject groups, as determined by chi-squared test for categorical variables and by non-parametric Kruskal–Wallis test for numerical variables. Apo, apolipoprotein; BMI, body mass index; HDL-C, high-density lipoprotein cholesterol; LDL-C, low-density lipoprotein cholesterol; Lp(a), lipoprotein(a); sdLDL-C, small dense low-density lipoprotein cholesterol; VLDL-C, very low-density lipoprotein cholesterol.

**Table 2:** Risk factors in women with established CHD compared with controls *.

Variable	Controls (*n* = 243)	CHD Cases (*n* = 107)	Percent Difference ^†^	*p* ^‡^
Standard Risk Factors				
Age, years	65.3 (12.2)	60.5 (17.2)	+7.4%	0.004
BMI, kg/m^2^	24.3 (5.4)	26.3 (7.9)	+8.2%	<0.001
Hypertension, %	16.5%	59.8%	+262.4%	<0.001
Diabetes, %	0.4%	25.2%	+6200%	<0.001
Current smoker, %	2.1%	10.3%	+390.5%	0.002
Lipids				
Total cholesterol, mg/dL	208.0 (47.0)	211.0 (72.5)	+1.4%	0.375
Triglycerides, mg/dL	111.0 (72.0)	144.0 (86.5)	+29.7%	<0.001
HDL-C, mg/dL	67.0 (23.0)	49.0 (23.5)	−26.9%	<0.001
Non-HDL-C, mg/dL	140.0 (48.5)	164.0 (88.5)	+17.1%	<0.001
Calculated LDL-C, mg/dL	116.0 (44.8)	129.6 (62.9)	+11.7%	0.017
Advanced Risk Factors				
Hs-C-reactive protein, mg/L	0.9 (2.1)	2.7 (4.4)	+200.0%	<0.001
Serum amyloid A, mg/L	3.9 (4.7)	5.2 (8.1)	+33.3%	0.007
Myeloperoxidase, pmol/L	183 (99)	377 (222)	+106%	<0.001
Direct LDL-C, mg/dL	116.0 (40.5)	135.0 (60.0)	+16.4%	0.003
sdLDL-C, mg/dL	26.0 (13.0)	37.0 (32.0)	+42.3%	<0.001
sdLDL-C, % of total LDL-C	23.0 (6.2)	24.8 (17.5)	+7.8%	<0.001
VLDL-C, mg/dL	23.0 (11.0)	25.0 (17.0)	+8.7%	0.079
ApoB, mg/dL	92.0 (29.5)	107.0 (45.0)	+16.3%	<0.001
Lp(a), mg/dL	10.6 (17.9)	18.0 (44.4)	+69.8%	0.009
Total apoA-I, mg/dL	175.4 (36.6)	160.8 (42.0)	−8.3%	<0.001
ApoA-I in preβ-1 HDL, mg/dL	13.1 (6.3)	15.2 (7.4)	+16.0%	0.005
ApoA-I in α-4 HDL, mg/dL	17.7 (5.6)	16.7 (4.5)	−5.6%	0.010
ApoA-I in α-3 HDL, mg/dL	24.3 (5.4)	23.3 (6.5)	−4.1%	0.094
ApoA-I in α-2 HDL, mg/dL	68.5 (15.3)	62.5 (19.6)	−8.8%	<0.001
ApoA-I in α-1 HDL, mg/dL	38.3 (18.0)	28.2 (20.2)	−26.4%	<0.001

* Data are expressed as median (interquartile range), except where percent is indicated. ^†^ CHD cases versus controls. ^‡^ *p* for trend across subject groups, as determined by chi-squared test for categorical variables and by non-parametric Kruskal–Wallis test for numerical variables. Apo, apolipoprotein; BMI, body mass index; HDL-C, high-density lipoprotein cholesterol; LDL-C, low-density lipoprotein cholesterol; Lp(a), lipoprotein(a); sdLDL-C, small dense low-density lipoprotein cholesterol; VLDL-C, very low-density lipoprotein cholesterol.

**Table 3:** Multivariate modeling in men.

	Odds Ratio *	SE	95% CI
Standard Lipid Model ^†^			
HDL-C	0.932	0.012	0.908, 0.954
Non-HDL-C	1.008	0.004	1.001, 1.015
Diabetes	126.242	137.504	22.211, 2422.739
Hypertension	3.922	1.135	2.236, 6.976
Smoking	3.659	2.632	0.858, 15.343
Standard Lipid Model Plus hsCRP ^‡^			
HDL-C	0.934	0.012	0.909, 0.956
Non-HDL-C	1.009	0.004	1.002, 1.016
Diabetes	107.950	117.569	19.062, 2073.068
Hypertension	3.826	1.115	2.172, 6.829
Smoking	3.726	2.680	0.874, 15.614
hsCRP	1.018	0.019	0.988, 1.070
Standard Model Plus hsCRP Plus Atherogenic Particles ^§^			
HDL-C	0.939	0.012	0.915, 0.962
Non-HDL-C	0.998	0.007	0.984, 1.012
Diabetes	122.297	134.677	21.078, 2383.910
Hypertension	3.622	1.071	2.038, 6.517
Smoking	3.953	2.877	0.918, 16.880
hsCRP	1.020	0.019	0.990, 1.072
Log sdLDL	2.558	1.485	0.823, 8.073
Log Lp(a)	1.488	0.249	1.070, 2.068
Standard Model Plus hsCRP Plus Atherogenic Particles Plus HDL Particles ^¶^			
HDL-C	0.837	0.031	0.775, 0.899
Non-HDL-C	1.005	0.008	0.989, 1.021
Diabetes	555.141	949.233	40.137, 36,420.222
Hypertension	4.550	1.505	2.404, 8.836
Smoking	5.575	4.679	1.045, 29.939
hsCRP	0.983	0.019	0.950, 1.032
Log sdLDL	1.213	0.886	0.288, 5.108
Log Lp(a)	1.446	0.268	1.004, 2.085
ApoA-I in preβ-1	1.137	0.049	1.046, 1.239
ApoA-I in α-1	1.043	0.036	0.974, 1.116
ApoA-I in α-2	1.099	0.030	1.044, 1.161
ApoA-I in α-3	0.898	0.044	0.814, 0.987
ApoA-I in α-4	0.899	0.043	0.817, 0.986

* Odds ratio is expressed per 1 mg/dL change, except for hypertension and smoking, which are expressed as “yes or no” and hsCRP, which is expressed per 1 mg/L change. ^†^ Standard lipid model, AUC: 0.856. ^‡^ Standard lipid model plus log hsCRP, AUC: 0.850. ^§^ Standard lipid model plus log hsCRP, log sdLDL-C, and log Lp(a), AUC 0.869. ^¶^ Standard lipid model plus log hsCRP, log sdLDL-C, log Lp(a), and apoA-I concentrations in all HDL particles, AUC: 0.913. Apo, apolipoprotein; CI, confidence interval; HDL-C, high-density lipoprotein cholesterol; hsCRP, high-sensitivity *C*-reactive protein; Lp(a), lipoprotein(a); sdLDL, small dense low-density lipoprotein; SE, standard error.

**Table 4:** Multivariate modeling in women.

	Odds Ratio *	SE	95% CI
Standard Lipid Model ^†^			
HDL-C	0.962	0.009	0.944, 0.980
Non-HDL-C	1.006	0.003	0.999, 1.013
Diabetes	27.323	28.708	5.263, 502.902
Hypertension	4.872	1.462	2.719, 8.842
Smoking	2.510	1.770	0.629, 10.415
Standard Lipid Model Plus hsCRP ^‡^			
HDL-C	0.965	0.009	0.946, 0.983
Non-HDL-C	1.006	0.003	0.999, 1.013
Diabetes	22.151	23.266	4.272, 407.569
Hypertension	4.818	1.458	2.675, 8.790
Smoking	2.261	1.597	0.570, 9.454
hsCRP	1.059	0.030	1.002, 1.122
Standard Model Plus hsCRP Plus Atherogenic Particles ^§^			
HDL-C	0.965	0.010	0.945, 0.983
Non-HDL-C	1.003	0.007	0.989, 1.016
Diabetes	21.355	22.514	4.078, 394.563
Hypertension	4.798	1.471	2.644, 8.826
Smoking	2.089	1.502	0.512, 8.941
hsCRP	1.064	0.031	1.005, 1.128
Log sdLDL	1.297	0.790	0.393, 4.321
Log Lp(a)	1.396	0.261	0.966, 2.018
Standard Model Plus hsCRP Plus Atherogenic Particles Plus HDL Particles ^¶^			
HDL-C	0.857	0.026	0.805, 0.907
Non-HDL-C	1.002	0.007	0.988, 1.017
Diabetes	20.673	23.305	3.347, 415.223
Hypertension	5.365	1.875	2.732, 10.805
Smoking	2.581	2.308	0.463, 15.194
Log hsCRP	1.078	0.034	1.014, 1.147
Log sdLDL	1.450	1.085	0.335, 6.371
Log Lp(a)	1.420	0.298	0.941, 2.150
ApoA-I in preβ-1	1.116	0.043	1.036, 1.205
ApoA-I in α-1	1.142	0.036	1.075, 1.218
ApoA-I in α-2	1.028	0.023	0.985, 1.074
ApoA-I in α-3	0.889	0.045	0.803, 0.980
ApoA-I in α-4	0.915	0.048	0.825, 1.013

* Odds ratio is expressed per 1 mg/dL change, except for hypertension and smoking, which are expressed as “yes or no” and hsCRP, which is expressed per 1 mg/L change. ^†^ Standard lipid model, AUC: 0.838. ^‡^ Standard lipid model plus log hsCRP, AUC: 0.842. ^§^ Standard lipid model plus log hsCRP, log sdLDL-C, and log Lp(a), AUC 0.844. ^¶^ Standard lipid model plus log hsCRP, log sdLDL-C, log Lp(a), and apoA-I concentrations in all HDL particles, AUC: 0.903. Apo, apolipoprotein; CI, confidence interval; HDL-C, high-density lipoprotein cholesterol; hsCRP, high-sensitivity *C*-reactive protein; Lp(a), lipoprotein(a); sdLDL, small dense low-density lipoprotein; SE, standard error; TG, triglycerides.

## Data Availability

The data presented in this study are available on request from the corresponding author. Restrictions may apply due to legal and ethical reasons.

## Supplementary Materials

The following supporting information can be downloaded at: https://www.mdpi.com/article/10.3390/nu17071182/s1, Table S1: Correlations in Men; Table S2: Correlations in Women.
