# Supplementary material for: High-Density Lipoprotein Particles, Inflammation, and Coronary Heart Disease Risk
Source: Nutrients. 2025 Mar 28;17(7):1182. doi: 10.3390/nu17071182 (PMC11990870; doi:10.3390/nu17071182)
Supplement: Supplementary file 1 [file nutrients-17-01182-s001.zip › nutrients-3485990-supplementary.pdf]

Supplement Table S1. Correlations in Men*

	α-1	Preβ-1	α-2	α-3	α-4	Log hsCRP	Log sdLDL	Log Lp(a)	HDL-C	Log nonHDL-C	SAA	log TG	TC
α-1		0.035	0.708	-0.206	0.128	-0.223	-0.377	-0.034	0.899	-0.261	-0.028	-0.475	0.098
Preβ-1	0.035		0.215	0.255	0.102	0.012	0.412	0.065	0.104	0.343	0.005	0.257	0.395
α-2	0.708	0.215		0.123	0.132	-0.172	-0.108	0.019	0.805	-0.103	0.028	-0.317	0.218
α-3	-0.206	0.255	0.123		0.426	-0.082	0.414	0.027	-0.055	0.302	-0.147	0.348	0.290
α-4	0.128	0.102	0.132	0.426		-0.15	0.123	-0.043	0.212	0.172	-0.119	0.116	0.257
Log hsCRP	-0.223	0.012	-0.172	-0.082	-0.15		0.031	-0.012	-0.261	0.078	0.545	0.133	-0.011
Log sdLDL	-0.377	0.412	-0.108	0.414	0.123	0.031		0.130	-0.334	0.850	-0.050	0.602	0.718
Log Lp(a)	-0.034	0.065	0.019	0.027	-0.043	-0.012	0.130		-0.021	0.136	0.028	-0.057	0.136
HDL-C	0.899	0.104	0.805	-0.055	0.212	-0.261	-0.334	-0.021		-0.221	-0.057	-0.544	0.178
Log nonHDL-C	-0.261	0.343	-0.103	0.302	0.172	0.078	0.850	0.136	-0.221		-0.061	0.487	0.898
SAA	-0.028	0.005	0.028	-0.147	-0.119	0.545	-0.050	0.028	-0.057	-0.061		0.001	-0.073
Log TG	-0.475	0.257	-0.317	0.348	0.116	0.133	0.602	-0.057	-0.544	0.487	0.001		0.276
TC	0.098	0.395	0.218	0.290	0.257	-0.011	0.718	0.136	0.178	0.898	-0.073	0.276	

*Spearman correlations. All correlation coefficients >0.20 are statistically significant ($P<0.05$).

HDL-C, high density lipoprotein cholesterol; hsCRP, high sensitivity C-reactive protein; Lp(a), lipoprotein(a); SAA, serum amyloid A; sdLDL, small dense low-density lipoprotein; TC, total cholesterol; TG, triglycerides

Supplement Table S2. Correlations in Women*

	α-1	Preβ-1	α-2	α-3	α-4	Log hsCRP	Log sdLDL	Log Lp(a)	HDL-C	Log nonHDL-C	SAA	log TG	TC
α-1		-0.007	0.610	-0.159	0.059	-0.357	-0.350	-0.043	0.903	-0.363	-0.084	-0.491	0.050
Preβ-1	-0.007		0.179	0.312	0.190	0.093	0.475	-0.021	0.039	0.365	0.001	0.333	0.435
α-2	0.610	0.179		0.249	-0.035	-0.184	-0.081	-0.043	0.708	-0.104	-0.047	-0.226	0.221
α-3	-0.159	0.312	0.249		0.394	0.025	0.389	-0.065	-0.031	0.318	-0.076	0.387	0.325
α-4	0.059	0.190	-0.035	0.394		0.002	0.212	-0.040	0.091	0.184	-0.026	0.215	0.251
Log hsCRP	-0.357	0.093	-0.184	0.025	0.002		0.161	0.111	-0.373	0.180	0.542	0.241	0.014
Log sdLDL	-0.350	0.475	-0.081	0.389	0.212	0.161		0.078	-0.350	0.860	0.039	0.615	0.741
Log Lp(a)	-0.043	-0.021	-0.043	-0.065	-0.040	0.111	0.078		-0.055	0.130	0.194	0.026	0.130
HDL-C	0.903	0.039	0.708	-0.031	0.091	-0.373	-0.350	-0.055		-0.359	-0.109	-0.572	0.095
Log nonHDL-C	-0.363	0.365	-0.104	0.318	0.184	0.180	0.860	0.130	-0.359		0.025	0.566	0.864
SAA	-0.084	0.001	-0.047	-0.076	-0.026	0.542	0.039	0.194	-0.109	0.025		0.090	-0.021
Log TG	-0.491	0.333	-0.226	0.387	0.215	0.241	0.615	0.026	-0.572	0.566	0.090		0.329
TC	0.050	0.435	0.212	0.325	0.251	0.014	0.741	0.103	0.095	0.864	-0.021	0.329	

*Spearman correlations. All correlation coefficients >0.20 are statistically significant (P<0.05).

HDL-C, high density lipoprotein cholesterol; hsCRP, high sensitivity C-reactive protein; Lp(a), lipoprotein(a); SAA, serum amyloid A; sdLDL, small dense low density lipoprotein; TC, total cholesterol; TG, triglycerides
